# On Imported and Domestic Human Papillomavirus Vaccines: Cognition, Attitude, and Willingness to Pay in Chinese Medical Students

**DOI:** 10.3389/fpubh.2022.863748

**Published:** 2022-05-12

**Authors:** Liangru Zhou, Baiyang Gu, Xiaoxue Xu, Yue Li, Pengxin Cheng, Yue Huo, Guoxiang Liu, Xin Zhang

**Affiliations:** School of Health Management, Harbin Medical University, Harbin, China

**Keywords:** HPV vaccine, immunization coverage, CVM, Tobit model, willingness to pay

## Abstract

This study aimed to analyze the cognition, attitude, and willingness to pay (WTP) for imported and domestic human papillomavirus (HPV) vaccines in Chinese medical students.

**Methods:**

Medical students in Eastern, Central and Western China were investigated. We used the HPV cognitive list to measure the cognition of participants and implemented contingent valuation method (CVM) to value WTP. Tobit model was used to analyze the factors associated with WTP.

**Results:**

The participants' average score for the 21 cognitive questions was 13.05 (±5.09). Among the participants, 60.82 and 88.01% reported that they would wish to be vaccinated and support the partners to be vaccinated. In addition, 92.54% (670) of the participants were willing to pay for HPV vaccines, at mean values (in RMB) of 1,689.80 (±926.13), 2,216.61 (±1190.62), and 3,252.43 (±2064.71) for imported bivalent, quadrivalent, and 9-valent vaccines, respectively, and at 910.63 (±647.03), 1,861.69 (±1147.80), and 2,866.96 (±1784.41) for their domestic counterparts, respectively. The increase in cognitive score has a positive effect on the WTP for imported vaccines (*P* < 0.05).

**Conclusions:**

Most of the participants were likewise willing to receive the HPV vaccines. Their perceptions of the HPV vaccines valent and origin may affect their willingness to be vaccinated and pay for the vaccines. Increasing awareness of the HPV vaccines and the inclusion of the HPV vaccines in a Medicare reimbursement policy or immunization program could increase the coverage of the HPV vaccine.

## Introduction

The human papillomavirus (HPV) vaccine was designed to protect humans from the risk of disease caused by HPV. When vaccinated, a vaccinated person can expect their immune system to respond to the virus, if exposed (1). Both males and females of appropriate age can be vaccinated with the quadrivalent or 9-valent vaccines.

The World Health Organization has stated that all three registered HPV vaccines, namely, the bivalent, quadrivalent, and 9-valent vaccines, have good safety, efficacy, and effectiveness (2). Studies have shown that HPV vaccination in 156 out of 179 countries has an incremental cost-effectiveness ratio of less than one time per capita GDP for saving one disability-adjusted life year (DALY), which is very cost-effective (3). The cost of cancer treatment is estimated to be reduced by approximately USD 12,400 for every quality-adjusted life years (QALY) received by adolescents and young adults in the United States receiving the HPV vaccine (4). Mo et al. pointed out that combined with the screening strategy of cervical cancer in mainland China, the vaccination of quadrivalent and 9-valent vaccines has been highly cost-effective (5).

At present, 110 countries and regions worldwide have included the HPV vaccine in their immunization programs and vaccinated the target population for free (6). However, the HPV vaccine has not been included in the medical insurance or immunization program in China. Residents need to purchase the HPV vaccine in full at their own expense. This hinders the coverage of HPV vaccines to a certain extent. The main problems encountered in the promotion of any new vaccine, especially those that require payment, are the public's response and the attitudes of different groups toward the purchase of vaccines (7, 8). Particularly in developing countries, high prices have always been a major obstacle to the introduction of HPV vaccines. Therefore, understanding the WTP for HPV vaccines of residents in developing countries is of great significance to the introduction and pricing of vaccines. Research on willingness to pay (WTP) for HPV vaccines has been carried out in Vietnam (9), Nigeria (10), Thailand (11) and other countries. However, evidence of WTP for HPV vaccines is still lacking in China. Our study aimed to provide more evidence on WTP for HPV vaccines from China and to explore factors associated with WTP for HPV vaccines.

## Materials and Methods

### Study Design and Implementation

We conducted an anonymous survey on medical students in Harbin Medical University, Hebei Medical University, and Chengdu University of Traditional Chinese Medicine, representing central, eastern, and western China, respectively, from November 2020 to March 2021. We used online forms to collect information via https://www.wjx.cn/. We applied the CVM to evaluate the WTP of each participant. Each respondent received 2RMB in cash as remuneration, which was paid through online payment. The Harbin Medical University School of Health Management & nstitutional Research Board approved the study protocol (HMUIRB20210006).

### Data Collection and Questionnaire Measures

The online form contained items on the basic information of respondents, the perceptions of the respondents about HPV infections and vaccines and their WTP. A total of 850 medical students were invited. Basic information included demographic information, such as sex, age, educational background, type of family residence, partner status, monthly consumption level, and health behaviors. We used HPV cognitive lists to assess the participants' knowledge of HPV infections and vaccines. The list contains 21 items, to which the participants responded either agree, unclear, or disagree. We compiled the 21 items from existing HPV cognition research, popular discussions on HPV vaccines in popular social media apps (Zhihu, WeChat, Weibo), and supplementary content through derived from pre-surveys and interviews. We mixed all cognitive content into a pool of cognitive items. Duplicate items were eliminated, and the items were further filtered to form the final list (**Table 2**). The score for this measure was the cumulative number of items answered correctly by the participant (1 point per correct response). The score ranged from 0 to 21 points, with higher scores indicating a higher likelihood of the participant having richer knowledge of HPV infections and vaccines. We also calculated the percentage of each item that was answered correctly to analyze the participants' perception of each knowledge item. Those who reported negative responses to the questions “Have you heard of HPV?” and “Have you heard of the HPV vaccine?” were not asked to answer this cognitive list. Notably, before responding to the above two questions related to vaccination attitudes, the respondents read the WHO introduction on HPV(Additional file 1) (12, 13). This approach was implemented to avoid the respondents from making rash decisions attributable to their lack of HPV knowledge when answering questions.

Respondents who expressed acceptance of the HPV vaccine answered the WTP questionnaire. We calculated WTP as the amount of money willing to spend on vaccines. We used the questions “If the HPV vaccine is included in the national immunization program and can be availed for free, would you be willing to be vaccinated?” and “If the HPV vaccine needs to be availed at your own expense, will you get yourself vaccinated?” to analyze respondents' attitude toward the HPV vaccine when it is provided for free and at their own expense. If the respondent was still unwilling to be vaccinated under the assumption of zero price, then the respondent was asked to report the reason. Those willing to pay for vaccination were asked about their views on the different full-dose prices of HPV vaccines.

**Figure 1:**
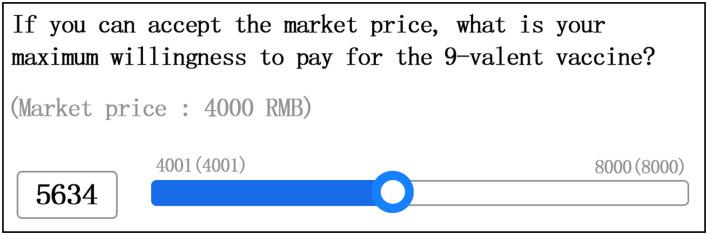
WTP for 9-valent HPV vaccine sliding price (example 1).

**Figure 2:**
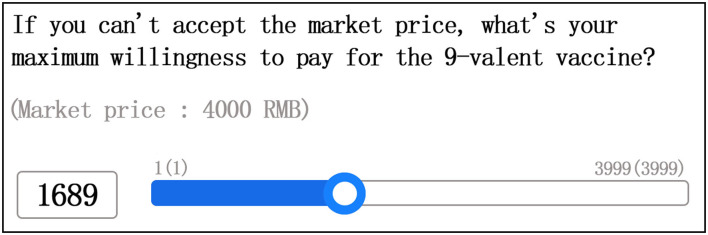
WTP for 9-valent HPV vaccine sliding price (example 2).

The contingent valuation method was used to obtain the respondents' WTP. To simulate the real-market environment, we adopted the iterative bidding game (IBG) methods (14) in our investigation of the participants' WTP. IBG is the guiding method of CVM and has been criticized for putting pressure on interviewees owing to repeated inquiries. To address the issues related to repeated price inquiry, we used sliding scale fees to ask the respondents' WTP for imported HPV vaccines. Participants directly slid the price ruler to mark their expected price. They could also directly fill in the expected price in the space to the left of the price ruler. We divided the range of the price ruler according to the responses to the market price. The price scale was divided into two parts based on respondents' response to the initial price, as shown in Figures 1, 2. The beginning and end price in the ruler fluctuated by 1 RMB according to the market price.

Given that only bivalent vaccines are available as domestic vaccines in China (15), and the other two valence vaccine types are not yet on the market, the WTP for domestic vaccines cannot provide the market price. Thus, we used the IBG method to ask those who were willing to be vaccinated with the domestic vaccine to slide the price ruler directly to mark the price. The price scale range was “1 to Import Vaccine market price ^*^ 2 RMB”.

We limited the vaccine valence types that the respondents could fill in by setting age limits. Females aged 9–26 years could answer the WTP for full valent vaccines, whereas females aged 27–45 years were only allowed to answer the WTP for bivalent and quadrivalent vaccines (16). In mainland China, the HPV vaccine is not yet recommended for males. But in the United States males were allowed to receive the quadrivalent/9-valent HPV vaccine. Males aged 9–26 years were allowed to answer the WTP for quadrivalent and 9-valent vaccines, whereas males aged 27–45 years could answer the WTP for 9-valent vaccine (17, 18).

### Statistical Analyses

Statistical analyses were performed using Stata Version 15 and Microsoft Excel Version 2016. We analyzed the participants' degree of knowledge reserve, vaccination attitudes, and WTP for different valence vaccines. The McNemar's test was used to compare whether there were differences in the acceptance rate of vaccines from different origins. Tobit model was used to analyze the influencing factors of WTP. Respondent who unwilling to pay for the vaccine, his WTP is recorded as 0, and there is a censorship of the lower limit of WTP. At the same time, limited by the scope of the WTP scale, WTP has an upper limit of censorship. Tobit model is suitable for analyzing dependent variable has upper or lower limit. *P* < 0.05 was considered significant.

## Results

### Participants' Characteristics

Table 1 summarizes the basic characteristics of the respondents. A total of 809 medical students were surveyed, including 475 female (58.71%) and 334 male (41.29%). There are 600 people aged 16–26 (74.17%), and 209 people aged 27–45 (25.83%).

**Table 1:** Demographic characteristics of participants.

**Characteristics**	***N* (%)**
Total	809 (100.00)
**Sex**	
Female	475 (58.71)
Male	334 (41.29)
**Age group**	
16–26	600 (74.17)
27–45	209 (25.83)
Educational background	
Undergraduate	419 (51.79)
Graduate and above	390 (48.21)
**Type of family residence**	
Urban	626 (77.38)
Rural	183 (22.62)
**Partner status**	
No partner	702 (86.77)
Have a partner	107 (13.23)
**Consumption level (Monthly)**	
≤2,000 RMB	469 (57.97)
>2,000 RMB	340 (42.03)
**Hours of exercise (Weekly)**	
≤3 h	681 (84.18)
>3 h	128 (15.82)

### Knowledge of HPV Infection, Related Diseases, and Prevention

Among the respondents, 751 (92.83%) had heard of HPV, and 728 (89.99%) had heard of the HPV vaccine. A total of 728 respondents answered the questions in the cognitive list. The average cognitive score was 13.05 (±5.09) points. The respondents maintained a high level of cognition of HPV infection, transmission, and vaccination population but reported insufficient awareness of post-vaccination. Most of them correctly replied that HPV could be sexually transmitted (86.81%) and that males and females contracted HPV (79.95%) (Table 2). Moreover, 60.85% of the respondents were aware that an HPV infection can be transmitted from the pregnant mother to the baby during pregnancy.

**Table 2:** Respondents' HPV cognitive list.

**Knowledge statement**	**Response**	**Correct (N/%)**	**Not sure (N/%)**	**Wrong (N/%)**
**Infection and transmission**				
Coitus is one of the main routes of HPV infection	True	632 (86.81)	71 (9.75)	25 (3.43)
There is no need to get the HPV vaccine as long as you have only one regular sexual partner	False	455 (62.50)	107 (14.70)	166 (22.80)
Both male and female can be infected with HPV	True	582 (79.95)	102 (14.01)	44 (6.04)
A woman infected with HPV is bound to develop cervical cancer	False	431 (59.20)	131 (17.99)	166 (22.80)
HPV infection can be passed from mother to child during pregnancy or during childbirth	True	443 (60.85)	217 (29.81)	68 (9.34)
HPV can be transmitted through indirect contact, such as underwear	True	350 (48.08)	175 (24.04)	203 (27.88)
Having HPV increases the chances of contracting HIV	False	100 (13.74)	178 (24.45)	450 (61.81)
A person may be infected with HPV virus without any symptoms	True	589 (80.91)	113 (15.52)	26 (3.57)
**Applicable population**				
People who have sex can also be vaccinated against HPV	True	569 (78.16)	118 (16.21)	41 (5.63)
Pregnant female are not recommended to receive HPV vaccine	True	443 (60.85)	234 (32.14)	51 (7.01)
If you have been infected with HPV, you don't need to get the HPV vaccine again	False	420 (57.69)	161 (22.12)	147 (20.19)
Male do not need to be vaccinated against HPV	False	404 (55.49)	197 (27.06)	127 (17.45)
**Post-vaccination**				
The HPV vaccine is harmful to health	False	350 (48.08)	159 (21.84)	219 (30.08)
The HPV vaccine causes an HPV infection	False	357 (49.04)	165 (22.66)	206 (28.30)
Female can not participate in cervical cancer screening after receiving HPV vaccine	False	506 (69.51)	98 (13.46)	124 (17.03)
To prevent HPV, you still need to use condoms if you have sex after getting the HPV vaccine	True	557 (76.51)	115 (15.80)	56 (7.69)
Female who have been vaccinated against HPV will not get cervical cancer	False	497 (68.27)	118 (16.21)	113 (15.52)
HPV vaccines available on the market can treat HPV infections	False	425 (58.38)	131 (17.99)	172 (23.63)
The higher the HPV vaccine order, the more viruses are prevented	True	505 (69.37)	142 (19.51)	81 (11.13)
It is meaningless to vaccinate other valence vaccines except 9 valence	False	445 (61.13)	132 (18.13)	151 (20.74)
The HPV vaccine approved by China was eliminated by other countries	False	440 (60.44)	142 (19.51)	146 (20.05)

Regarding the applicable population of HPV vaccine, 78.16% of the participants knew that males can also get the HPV vaccine, 60.85% knew that vaccination is not be recommended for pregnant females, and 55.49% knew that males need to receive the HPV vaccine. More than half (57.69%) of the respondents knew that those who have contracted HPV could also be vaccinated.

Of the post-vaccination part, 30% did not know that they still need cervical cancer screening after HPV vaccination. About 30% could not clearly recognize that the HPV vaccine reduces the risk of cervical cancer but not eliminate it. Nearly 40% of the respondents hold the erroneous view that 9-valent has the highest protective effect, and that vaccination with other valence types is meaningless if the 9-valent vaccine cannot be vaccinated. Meanwhile, 39.56% reported misinformation that China's vaccines are obsolete abroad, and 30% had the false view that an HPV vaccine is harmful to the health and leads to HPV infection.

### Attitudes on HPV Infection and Prevention

We investigated the attitudes of respondents themselves and their partners toward HPV. A total of 60.82% of the people reported that they would like to be vaccinated, 30.90% of the respondents said that they did not have the willingness to be vaccinated at this stage, and a total of 8.28% were unwilling to be vaccinated. Table 3 summarizes the results.

**Table 3:** Respondents ' attitudes toward HPV vaccination.

**Vaccination attitudes**	***N***	**%**
**Respondents' vaccination attitudes**		
Willing to be vaccinated	492	60.82
May be vaccinated in the future	250	30.90
Unwilling to be vaccinated	67	8.28
**Vaccination attitudes toward partners/future partners**		
Support	712	88.01
It doesn't matter	88	10.88
No support	9	1.11

### Willingness to Pay for HPV Vaccines

Excluding 67 people (8.28%) who were unwilling to receive the HPV vaccine, a total of 742 people filled out the WTP questionnaire. Under the condition that the HPV vaccine is contained in the national immunization program, 724 (97.57%) respondents indicated their willingness to be vaccinated. The reluctance of the remaining 18 (2.43%) respondents was due to the unclear complications and low confidence in free vaccines.

In the case of the HPV vaccine charges, 670 (92.54%) of the respondents were willing to be vaccinated. The 54 (7.46%) who were unwilling to pay for the vaccine stated the following reasons other than the price: “I think it is unnecessary,” “After vaccination, it is not once and for all,” “HPV can be prevented by condoms, and there is no need to spend so much money on vaccination,” “Vaccination is painful,” and “Lack of authoritative evidence for long-term side effects.”

**Figure 3:**
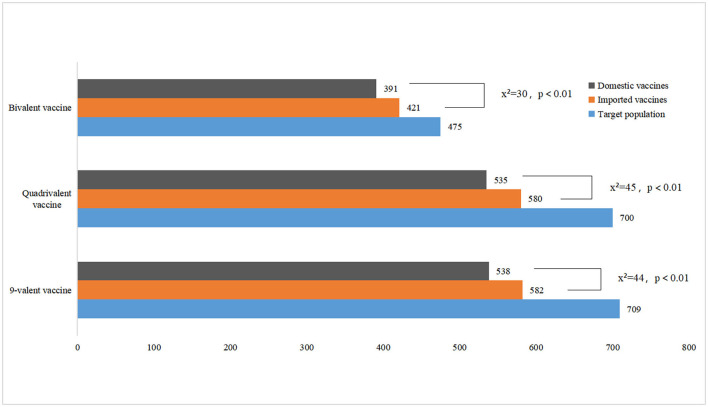
Respondents' willing to pay for HPV vaccination.

**Figure 4:**
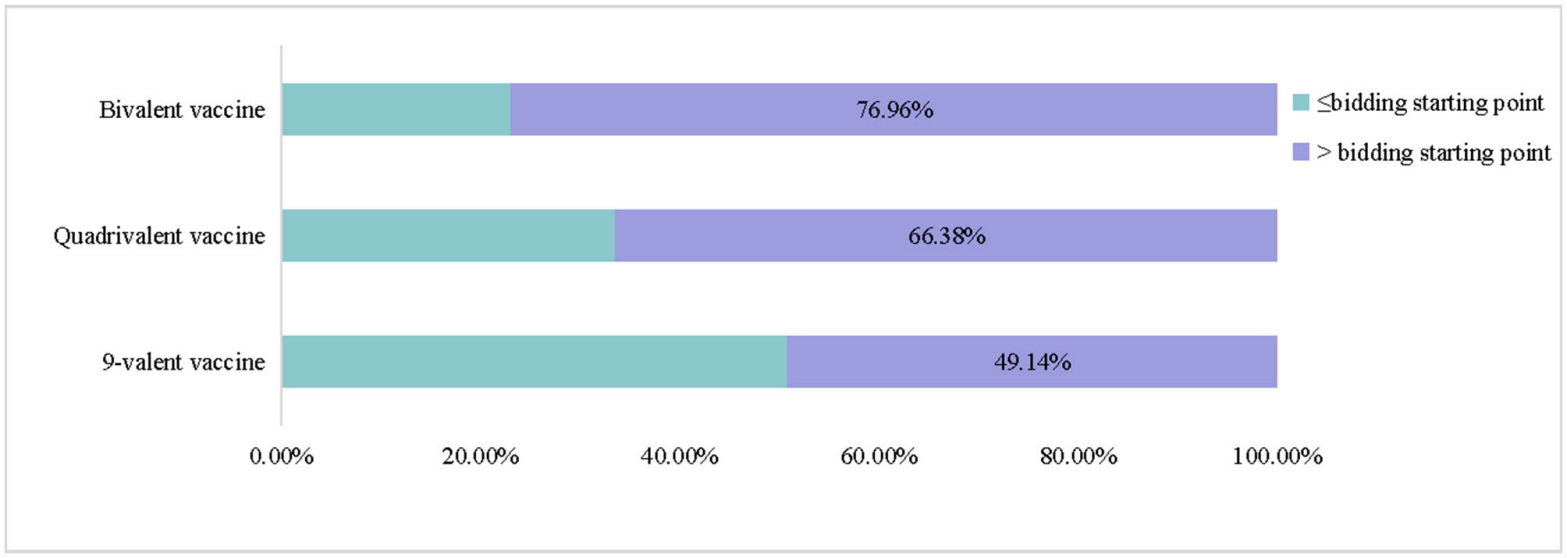
Distribution of respondents' WTP for different valences vaccines.

Figure 3 presents the number of people willing to pay for HPV vaccines. The number of people willing to pay for domestic vaccines of various valence was lower than that of imported vaccines (*P* < 0.01). Respondents whose WTP for imported vaccines exceeded the current market price of 1,740 RMB accounted for 76.96% of the target population for the bivalent. Respondents with a WTP higher than 2,469 RMB accounted for 66.38% of the total population of those willing to be vaccinated with a quadrivalent vaccine. Respondents with a WTP higher than 4,000 RMB accounted for 49.14% of the target population of the 9-valent vaccine (Figure 4).

Table 4 gives the WTP values in RMB for imported and domestic HPV vaccines. The results revealed that the average WTP of the vaccines was lower than the market price. Moreover, the WTP for domestic vaccines was lower than that for imported vaccines, and the gap in WTP for bivalent vaccines was the largest.

**Table 4:** WTP for imported and domestic HPV vaccines, full dose.

**Willingness to pay**	**Bivalent (1740RMB)**	**Quadrivalent (2469 RMB)**	**9-valent (4000 RMB)**
	**Mean±SD (RMB)**	**Mean ±SD (RMB)**	**Mean ±SD (RMB)**
WTP for imported vaccines	1689.80 ± 926.13	2216.61 ± 1190.62	3252.43 ± 2064.71
≤ Bidding starting point	604.34 ± 400.89	944.08 ± 614.64	1603.23 ± 1033.79
> Bidding starting point	2150.05 ± 578.31	2941.60 ± 603.20	4862.23 ± 1093.44
WTP for domestic vaccines	910.63 ± 647.03	1861.69 ± 1147.80	2866.96 ± 1784.41

### Multivariate Analysis

The dependent variables of the multiple regression include respondents who are unwilling to pay for the vaccine (Table 5). Rural household registration, graduate degree and above had a negative effect on the WTP for various vaccines (*P* < 0.05). The increase in cognitive score has a positive effect on the WTP for imported vaccines (*P* < 0.05). Female's WTP for domestic quadrivalent and imported 9-valent HPV vaccines was higher than that of male (*P* < 0.05). After controlling for the fact that WTP has upper and lower limit, female is expected to increase the WTP by 387.23 and 612.72 yuan, ceteris paribus.

**Table 5:** Tobit model of WTP for HPV vaccines.

**Characteristics**	**Bivalent HPV vaccine**		**Quadrivalent HPV vaccine**		**9-valent HPV vaccine**	
	**Imported**	**Domestic**	**Imported**	**Domestic**	**Imported**	**Domestic**
	**Coef (95%CI)**	**Coef (95%CI)**	**Coef (95%CI)**	**Coef (95%CI)**	**Coef (95%CI)**	**Coef (95%CI)**
27–45 year	−98.24	70.05	−63.59	90.73	–	–
	(−348.71, 152.23)	(−99.33, 239.42)	(−384.6, 257.42)	(−213.75, 395.22)	-	–
Female	–	–	26.51	387.23^*^	612.72^**^	195.55
	–	–	(−373.03, 426.06)	(12.50, 761.96)	(166.96, 1058.47)	(−194.89, 585.98)
Graduate and above	−140.15^**^	−59.86^*^	−150.86^**^	−68.17	−383.81^***^	−196.89^**^
	(−228.89, −51.41)	(−118.65, −1.07)	(−252.29, −49.43)	(−162.54, 26.21)	(−553.17, −214.44)	(−344.15, −49.62)
Rural	−341.96^**^	−191.36^*^	−297.23^*^	−438.45^***^	−734.41^**^	−596.24^**^
	(−569.06, −114.86)	(−346.00, −36.71)	(−573.4, −21.05)	(−700.94, −175.96)	(−1,202.5, −266.33)	(−1,000.13, −192.35)
Have partner	194.93	186.38	27.43	168.46	−61.02	−454.24
	(−136.48, 526.35)	(−32.47, 405.22)	(−322.64, 377.5)	(−157.80, 494.71)	(−855.12, 733.08)	(−1110.01, 201.53)
Exercise hours>3 h	343.13^*^	166.55	315.05^*^	273.84	−407.44	183.51
	(73.50, 612.77)	(−13.71, 346.81)	(8.76, 621.34)	(−10.57, 558.25)	(−995.41, 180.54)	(−329.62, 696.64)
Cognitive score	35.58^**^	0.33	45.25^***^	−7.23	71.56^***^	−13.45
	(15.14, 56.02)	(−13.96, 14.63)	(21.83, 68.67)	(−29.97, 15.51)	(32.6, 110.51)	(−48.31, 21.41)
>2,000 RMB	118.57	42.56	156.82	125.06	233.86	232.10
	(−92.19, 329.35)	(−86.45, 180.60)	(−91.78, 405.43)	(−113.09, 363.21)	(−203.93, 671.66)	(−148.45, 612.65)
_cons	1587.19^***^	849.31^***^	1,984.90^**^	1154.80	3,693.26^***^	4,173.61^***^
	(744.15, 2430.23)	(287.90, 1410.72)	(572.99, 3396.79)	(−163.88, 2473.47)	(1938.73, 5447.78)	(2676.34, 5670.88)

*CI: confidence interval*;

* *P < 0.05*;

** *P < 0.01*;

*** *P < 0.001; -:not applicable*.

## Discussion

Our research reported respondents' awareness of HPV transmission, pathogenesis, vaccination, and the applicable population. The results showed that Chinese medical students were no strangers to HPV or its vaccines. Almost all (92.83%) of the respondents had heard of HPV. This result is slightly lower than that of an Italian study of nursing students (96%) (19). It is also slightly lower than that in a study in Fujian, China, in which 96.1% of medical students said they “know HPV”(20). In our study, 89.99% of the respondents reported that they had heard of the HPV vaccine. In contrast to medical students in southern India, only 59.7% of whom reported HPV cognition (21), Chinese medical students may be more aware of HPV vaccines.

Our participants lacked or had poor knowledge of HPV's transmission channels. Most of them knew that HPV is a sexually transmitted virus and that both males and females can be infected (>80%). Nonetheless, they did not know that HPV can be transmitted through mother-to-child transmission (60%) and indirect contact (48.08%). Moreover, 20% of them did not understand that males need to be vaccinated against HPV. People often consider HPV vaccines as cervical cancer vaccines. Once a vaccine is labeled as a sex vaccine, more effort is needed to correct misconceptions.

Males can directly benefit from HPV vaccination. A meta-analysis showed that vaccination with a quadrivalent vaccine can reduce the incidence of genital warts in boys (22). In our interview, there were male respondents who believed that they were not suitable to participate in the survey. This view reflected the prevailing misconceptions in society (23). In 2019, the WHO Strategic Advisory Group pointed out that the current supply of HPV vaccines is limited and called on countries to suspend the implementation of HPV vaccination strategies regardless of gender and age groups until all countries have equitable access to vaccine supplies (24). Only female vaccination is recommended in the clinical application of HPV vaccines (25). However, countries such as the Canada have begun to implement “sex-neutral” immunization programs (26).

The willingness to be vaccinated and to support the partners to be vaccinated tended to paint an optimistic trend. Only 8.28% of the participants clearly indicated their unwillingness to receive the HPV vaccine, and 1.11% did not support the partners to be vaccinated. Our results showed that 60.82% of the respondents were willing to be vaccinated. This result is similar to that in studies in Italian nursing students (65.3%) (19) and medical students in India (67.8%) (27). The results of the Tobit model show the importance of cognitive score to improve HPV vaccination coverage. It is very important to guide the interviewees to hold a positive view of HPV vaccine by popularizing HPV vaccine knowledge.

At present, there are bivalent domestic vaccines in China to replace imported bivalent vaccines. A randomized clinical experimental study in China showed that the domestic bivalent HPV vaccine has a high protection efficacy against uterine cancer (28). However, our participants reported a preference for imported vaccines. The imported HPV vaccine was approved by the US FDA in 2006 and has more than ten years of clinical use experience. In contrast, the first domestically produced HPV vaccine in China was launched at the end of 2019. From the perspective of vaccine quality, many people have expressed a preference for imported vaccines.

Crowd psychology may be at play in the choice of the 9-valent vaccine, instead of a full understanding of the differences between valence types. From the recommended age group of the 9-valent HPV vaccine, it is ideal for those entering puberty. However, owing to the shortage of imported 9-valent vaccines in the Chinese market, a large number of females are aspiring to be vaccinated with this type, which has further pushed up the market demand. Indeed, the Chinese public has called for high-level evidence to clarify the advantages of domestic and imported vaccines. Clinical trials and health economic evaluation methods can provide more evidence in terms of vaccine safety, effectiveness, and resource allocation efficiency. Meanwhile, more publicity and promotion are needed to expand public understanding of HPV and the different valence vaccines.

Our findings on WTP indicated that most medical students were willing to pay for the HPV vaccine. However, under the premise of free vaccinations, 18 respondents expressed an unwillingness to receive the HPV vaccine for safety and other reasons. When it needs to be paid, the proportion of the respondents who were unwilling to be vaccinated increased from 2.43 to 7.46%. In China, the HPV vaccine is not included in the national immunization program. Many people refuse or postpone vaccination because they cannot pay for the vaccine. A study conducted in Hong Kong showed that 67.60% of doctors and 70.50% of nurses find HPV vaccines expensive (29). China's immunization program and implementation of insurance reimbursement policies for HPV vaccines will help increase the HPV vaccination rate. At present, select regions in China have included HPV vaccines in their insurance reimbursements. In Guizhou, the first HPV vaccine is free and subsequent shots can be paid on balance via personal insurance (30, 31). Assuming that those who are willing to pay less than the bid price will give up vaccination, the vaccination rate of bivalent vaccines at the current price was about 76%, whereas that of quadrivalent vaccines was <70%, and that of the 9-valent vaccine was <50%. Reducing the price of vaccines on the market or promoting the launch and pricing of domestic vaccines can increase willingness to be vaccinated.

The WTP values for imported vaccines were all below the market price. The willingness to import value for 9-valence vaccines was also far lower than the market price. At present, the import price of 9-valent vaccines is deemed to be too high. The respondents reported having limited ability to pay, especially the higher fees for the imported 9-valent vaccines. Meanwhile, bivalent domestic vaccines are consistent with the market price. Domestic quadrivalent and 9-valent vaccines are not yet available; their pricing may refer to our evidence. Nonetheless, the respondents were willing to pay more for imported vaccines and less willing to pay for domestic vaccines. Specifically, the payment willingness for domestic bivalent vaccines was far lower than that for imported bivalent vaccines. This may be because China has already launched a domestic bivalent vaccine, and the respondents already have an anchor price. The bivalent HPV vaccine produced in China costs 329 RMB per vial, about half the price of the imported bivalent vaccine.

Our research provides evidence on Chinese medical student' WTP for HPV vaccines and on HPV awareness and vaccination attitudes. The published studies have involved parents of adolescents (8, 10, 11), and our study provides evidence for the HPV vaccine WTP in medical students. In addition to being the beneficiaries of vaccination, medical students are also important personnel to promote HPV vaccine immunization in the future. Paying attention to medical students' knowledge and attitudes toward HPV vaccine will help accelerate the coverage of HPV vaccine.

The following limitations must be considered when interpreting our findings. First, the respondents' WTP for vaccines may be underestimated owing to the price scale cap. Second, this study may be theoretically underrated given that the nationally produced quadrivalent and 9-valent HPV vaccines are not yet approved and permitted for distribution.

## Conclusions

Most of the respondents were willing to receive the HPV vaccine. The number of medical students who were willing to be vaccinated with domestically produced vaccines was lower compared with the trend for imported vaccines. Medical students' understanding of HPV vaccine valences, place of production, safety, and effectiveness need to be improved. Meanwhile, most of them were willing to pay for the HPV vaccine. Their perceptions of HPV vaccine valences and origin may affect their willingness to be vaccinated and pay for the vaccine. Government departments should fully consider the evidence regarding WTP when pricing vaccines. Medical insurance reimbursement policies or immunization program plans can eliminate the price barrier of vaccination to a certain extent, thus enhancing vaccination rates and protecting a larger population against HPV.

## Data Availability Statement

The raw data supporting the conclusions of this article will be made available by the authors, without undue reservation.

## Ethics Statement

The studies involving human participants were reviewed and approved by Harbin Medical University School of Health Management institutional Research Board (HMUIRB20210006). The patients/participants provided their written informed consent to participate in this study.

## Author Contributions

LZ proposed and initiated this study. LZ and BG wrote the first draft of the manuscript. XZ, XX, and GL designed the project and oversaw the analysis and manuscript writing. YL, PC, BG, and YH drawn figures in the manuscript. YL and BG inserted tables in the manuscript. GL supervised the entire project. All co-authors provided feedback during the design and interpretation of the project. They also contributed to revisions of the manuscript. All authors read and approved the final manuscript.

## Conflict of Interest

The authors declare that the research was conducted in the absence of any commercial or financial relationships that could be construed as a potential conflict of interest.

## Publisher's Note

All claims expressed in this article are solely those of the authors and do not necessarily represent those of their affiliated organizations, or those of the publisher, the editors and the reviewers. Any product that may be evaluated in this article, or claim that may be made by its manufacturer, is not guaranteed or endorsed by the publisher.
